# Association of 25-hydroxyvitamin D with sex hormones and body composition in Chinese older adults

**DOI:** 10.3389/fendo.2025.1714445

**Published:** 2025-11-17

**Authors:** Xinyu Miao, Cong Ma, Zhaoyan Gu, Shuangtong Yan, Yanping Gong, Guogang Xu

**Affiliations:** 1 Department of Endocrinology, The Second Medical Center & National Clinical Research Center for Geriatric Diseases, Chinese People's Liberation Army (PLA) General Hospital, Beijing, China; 2 Health Management Institute, The Second Medical Center & National Clinical Research Center for Geriatric Diseases, Chinese People's Liberation Army (PLA) General Hospital, Beijing, China

**Keywords:** vitamin D, sex hormones, body mass index, body composition, older adults

## Abstract

**Background:**

Evidence from several studies suggests that vitamin D deficiency is associated with reduced testosterone levels. However, evidence regarding the relationships between serum 25-hydroxyvitamin D [25-(OH)D] and sex hormones remains inconsistent. This study focuses on older adults, aiming to investigate the associations of 25-(OH)D with sex hormones and body composition.

**Methods:**

A cross-sectional study was conducted, enrolling 2472 older adults (1406 males; 1066 females) aged 60 years or older who underwent routine health examinations at the Chinese PLA General Hospital in 2018 and 2019. Anthropometric measurements, biochemical characteristics, sex hormones, body composition, and chronic comorbidities were compared after stratification by 25-(OH)D levels. Multiple linear regression and logistic regression analyses were performed to explore the relationships of 25-(OH)D status with sex hormones and body composition.

**Results:**

Among the older adults in this study, the proportion of those with insufficient/deficient vitamin D was 87.4% (1229/1406) in males and 91.8% (979/1066) in females. By 25-(OH)D stratification (from sufficiency to deficiency), males showed increasing trends in body mass index (BMI), waist circumference (WC), body fat percentage, muscle mass and basic metabolic rate, smoking rate, triglycerides (TG), parathyroid hormone (PTH), and prolactin (PRL), along with decreasing trends in albumin (Alb), serum calcium (Ca), high-density lipoprotein cholesterol (HDL-C), serum creatinine (Cr), aspartate aminotransferase (AST) and testosterone; females exhibited similar trends, with additional decreases in hemoglobin (Hb), luteinizing hormone (LH), and follicle-stimulating hormone (FSH) (all P<0.05). In males, a 10 ng/ml decrease in 25-(OH)D was associated with a -0.95 (-1.46, -0.45) nmol/L change in T levels and a 26% higher risk of hypogonadism (defined as total testosterone <12 nmol/L), though these associations disappeared after adjusting for age and BMI. For every 10 ng/ml decrease in 25-(OH)D, body fat percentage, muscle mass, and basal metabolic rate increased in both older males and females. After adjusting for confounding factors, only body fat percentage increased by 0.30 (0.00, 0.59) % in older males.

**Conclusion:**

In elderly men, 25-(OH)D levels correlated positively with total testosterone, but this correlation may be BMI-dependent. Additionally, as 25-(OH)D levels decreased, body fat percentage increased in this group, suggesting a potential mediating role of increased BMI/adiposity in the vitamin D-testosterone relationship.

## 1 Introduction

Vitamin D deficiency is a significant public health issue, with a high prevalence among older adults (>60 years old). Epidemiological studies have demonstrated that the global prevalence of vitamin D deficiency (defined as a serum 25-(OH)D level < 20 ng/mL) among older adults is as high as 58.7% to 90.3% (1–3). Therefore, the series of systemic changes and health issues associated with vitamin D deficiency in the elderly population deserves attention. Vitamin D plays a pivotal role in regulating calcium-phosphorus metabolism and maintaining the musculoskeletal system; moreover, most tissues and cells in the body express vitamin D receptors, and various roles of vitamin D have thus been investigated (4–6). Vitamin D receptors (VDR) and vitamin D-metabolizing enzymes are widely expressed in the male reproductive tract (7). Both animal and human studies indicate that vitamin D may also regulate androgen levels, particularly testosterone in males (8).

However, the relationship of serum 25-(OH)D status with gonadal hormone levels remains notably inconsistent. Some studies found that serum 25-(OH)D levels positively correlated with total testosterone levels in men (9–11), In contrast, other studies have failed to detect an association between 25-(OH)D and total testosterone levels (12, 13), or have demonstrated no significant independent association after adjustment for confounders such as body mass index (BMI), or health and lifestyle factors (14, 15). Moreover, in older adults, who exhibit physiological declines in sex hormone levels and a high burden of comorbidities, the specific pattern of association between vitamin D and sex hormones remains insufficiently validated. Therefore, we investigate the association of 25-(OH)D with sex hormones in older men and women to provide additional evidence regarding the impact of vitamin D deficiency on sex steroids in the elderly population.

Sex hormones, particularly testosterone and estradiol, are not only important for maintaining sexual function, but also crucial for preserving muscle mass and preventing excessive body fat accumulation (16, 17). Separately, a majority of studies have supported an inverse association between vitamin D levels and body fat percentage—i.e., lower vitamin D levels correlate with higher body fat percentage (18–21). A deficient vitamin D status could promote the process of adipogenesis, which may potentially result in increased adiposity (22). However, two key points remain to be clarified in the elderly population: the correlation between vitamin D and body composition, and whether vitamin D deficiency-related changes in sex hormones contribute to this relationship. This study hypothesizes that 25-(OH)D levels are negatively associated with body fat percentage, and that hypogonadism (low testosterone levels) related to vitamin D deficiency may be involved in this association.

## 2 Materials and methods

### 2.1 Study population

Participants were enrolled from the Department of Health Medicine, the Second Medical Center of Chinese PLA General Hospital, who underwent physical examinations between January 2018 and December 2019. Participants were excluded if they were aged < 60 years, had acute or chronic inflammatory diseases, heart failure, renal failure, or tumors, were taking vitamin D supplements or drugs that affect gonadotropins, had incomplete information on gonadotropins, or were unable to provide informed consent. Finally, a total of 2472 subjects were included in the study (see **Supplementary Figure 1**). All subjects signed an informed consent form. The study protocol was approved by the Medical Ethics Committee of Chinese PLA General Hospital.

### 2.2 Anthropometric and biochemical measures

All individuals underwent clinical examinations and anthropometric measurements conducted by experienced medical teams. Smoking and drinking status were collected via a brief questionnaire. Histories of diagnosed diseases were obtained from hospital medical records and confirmed through medical record review. Venous blood was collected from all subjects following an overnight fast, in accordance with the quality control and testing standards of the Clinical Laboratory of the Chinese PLA General Hospital. T, E_2_, P, LH, FSH, and PRL were detected using a fully automatic chemiluminescent immunoassay analyzer (Centaur instrument, Siemens AG, USA). Hypogonadism was defined as a total T level < 12.0 nmol/L (23, 24). Serum PTH was determined using an electrochemiluminescent immunoassay analyzer (Roche Group, Germany). HbA1c was measured via high-performance liquid chromatography (HLC-723G8, TOSOH, Japan). Alb, FPG, lipid profile, Cr, ALT, AST, Ca, P, UA, and CRP were detected using an automatic biochemical analyzer (Cobas c 702, Roche, Switzerland). Hb was detected with a fully automatic hematology analyzer X9000 (Sysmex, Japan). All experimental methods and procedures adhered to standardized laboratory protocols and guidelines. Serum 25-(OH)D levels were measured using a chemiluminescent immunoassay (CLIA) with a DiaSorin LIAISON^®^ analyzer (DiaSorin Inc, MN, USA), employing the direct competitive immunoassay LIAISON^®^ 25(OH) Vitamin D TOTAL Assay. Vitamin D status was categorized based on 25-(OH)D levels as follows: deficiency, < 20 ng/ml; insufficiency, ≥ 20 to < 30 ng/ml; and sufficiency, ≥ 30 ng/ml (5).

### 2.3 Body composition assessment

Body composition measurements were performed using a body composition analyzer manufactured by SELVAS, which has obtained “CE0123” certification in accordance with the Medical Device Directive (MDD/CE; 93/42/EEC). The analyzer is also certified to comply with the production and quality specifications of EN ISO 13485. It uses bioelectric impedance analysis (BIA), a reliable, safe, and non-invasive method for assessing individual body components. Anthropometric data were collected, and individual body compartments as well as basal metabolic rate were determined using this analyzer. All participants were informed of the contraindications for body composition analysis and confirmed that they had no such contraindications. Additionally, all participants maintained an overnight fast of at least 8 hours before the measurement (with measurements conducted in the morning).

### 2.4 Statistical analysis

Data were analyzed using SPSS Version 24.0 and R software. Normally distributed data are expressed as mean ± standard deviation (SD), non-normally distributed data as medians and quartiles, and categorical data as frequency (percentage). Comparisons of indicators across 25-(OH)D stratification groups were performed using one-way analysis of variance (ANOVA), while comparisons between groups with non-normal distribution were performed using the non-parametric Mann-Whitney U test. The chi-square test was used to compare differences in categorical data. The 25-(OH)D association with sex hormones was analyzed using multiple linear regression. Multivariate logistic regression was applied to investigate the association between 25-(OH)D and hypogonadism. The confounding factors for adjustment were based on the results of univariate analysis. Correlation coefficient plots were used to visualize the correlations between vitamin D and testosterone, body fat percentage, muscle mass, and basal metabolic rate in older men and women. A P-value < 0.05 was considered statistically significant.

## 3 Results

### 3.1 Comparison of anthropometric, biochemical characteristics, sex hormones, body composition and chronic comorbidities in elderly men stratified by 25-(OH)D levels

In this study, among older adults, the proportion of those with insufficient/deficient vitamin D was 87.4% (1229/1406) in males and 91.8% (979/1066) in females. Their average ages were 64.96 ± 4.66 years and 65.54 ± 4.85 years, respectively. After stratification by 25-(OH)D levels: sufficiency (≥30 ng/ml), insufficiency (20–<30 ng/ml), deficiency <20 ng/ml), the following trends were observed in elderly men as vitamin D levels decreased:

BMI, WC, WHR, smoking rate, TG, and PTH increased (P<0.05); Alb, Ca, HDL-C, Cr, and AST decreased (P<0.05). In sex hormones, serum PRL exhibited an increasing trend, while T declined (P<0.05). Additionally, body fat percentage, muscle mass and basic metabolic rate increased with decreasing 25-(OH)D levels (P<0.05). No significant differences were observed in the proportions of individuals with hypertension, diabetes, or coronary heart disease (CHD), as shown in **Table 1**.

**Table 1:** Comparison of key characteristics in elderly men stratified by 25-(OH)D levels.

25-(OH)D	Sufficiency (≥30 ng/ml), n=177	Insufficiency (20~30 ng/ml), n=695	Deficiency (<20 ng/ml), n=534	P-value
Age (years)	65.16 ± 4.56	64.94 ± 4.57	64.93 ± 4.79	0.831
BMI (kg/m^2^)	24.94 ± 3.10	25.50 ± 2.95	26.02 ± 3.15	<0.001
WC (cm)	90.98 ± 9.37	92.56 ± 8.89	93.99 ± 8.99	<0.001
WHR	0.95 ± 0.05	0.96 ± 0.05	0.97 ± 0.05	0.002
Smoke (%)	50 (28.2%)	221 (31.8%)	211 (39.5%)	0.009
Drink (%)	104 (58.8%)	427 (61.4%)	313 (58.6%)	0.139
T (nmol/L)	15.78 [12.34,19.47]	14.93 [11.73,19.03]	14.55 [11.46, 18.25]	0.043
E_2_ (pmol/L)	101.80 [80.51,126.85]	101.30 [78.03,126.93]	97.10 [76.75, 120.88]	0.117
LH (U/L)	7.21 [5.03,9.77]	6.97 [5.00,9.27]	7.00 [5.13, 9.20]	0.713
FSH (U/L)	8.72 [6.33, 13.82]	9.07 [6.57, 12.94]	8.67 [6.33, 12.93]	0.852
P (nmol/L)	0.26 [0.16, 0.50]	0.23 [0.16, 0.44]	0.21 [0.16, 0.44]	0.372
PRL (u;g/L)	10.92 [8.03,16.11]	11.73 [8.52,16.06]	13.12 [8.67, 17.30]	0.012
Ca (mmol/L)	2.32 ± 0.10	2.32 ± 0.09	2.28 ± 0.09	<0.001
PTH (pg/ml)	38.29[31.28,47.48]	41.50[33.29,50.16]	45.39[36.65,56.34]	<0.001
FPG (mmol/L)	5.65 [5.20,6.48]	5.68 [5.20,6.65]	5.64 [5.18, 6.68]	0.811
HbA1c (%)	5.90 [5.60,6.30]	6.00 [5.70,6.50]	6.00 [5.70,6.70]	0.107
TC (mmol/L)	4.30 [3.65,5.07]	4.46 [3.80,5.13]	4.22 [3.64,4.97]	0.016
LDL-C (mmol/L)	2.88 [2.26,3.59]	3.06 [2.36,3.72]	2.88 [2.23,3.56]	0.015
TG (mmol/L)	1.29 [0.97,1.77]	1.40 [1.02,1.95]	1.50 [1.06,2.13]	0.006
HDL-C (mmol/L)	1.23 [1.06,1.47]	1.19 [1.01,1.43]	1.10 [0.94,1.30]	<0.001
UA (mmol/L)	360.00 [320.50,405.00]	364.00 [315.00,421.00]	3522.50 [305.25,407.00]	0.032
Cr (u;mol/L)	78.00 [69.00,86.50]	76.00[68.00,84.00]	74.00[67.00,82.00]	0.001
ALT (U/L)	19.10 [13.95, 26.55]	19.40 [14.70, 25.60]	18.00 [13.70,25.05]	0.076
AST (U/L)	19.30 [15.40, 23.35]	18.20 [15.30, 21.90]	17.00 [14.62, 20.70]	<0.001
Alb (g/L)	45.26 ± 2.99	45.22 ± 3.11	44.31 ± 3.50	<0.001
Hb (g/L)	148.08 ± 13.04	148.46 ± 12.09	147.53 ± 13.28	0.402
CRP (mg/dl)	0.09 [0.05,0.18]	0.09 [0.04,0.20]	0.09 [0.05,0.19]	0.953
Diabetes (%)	40 (22.6%)	173 (24.9%)	144 (27.0%)	0.194
Hypertension (%)	71 (41.5%)	294 (43.7%)	255 (48.4%)	0.156
CHD (%)	16 (9.0%)	64 (9.2%)	45 (8.4%)	0.297
Body fat percentage (%)	24.30 [20.90, 27.00]	25.30 [22.30, 27.90]	25.70 [22.70, 28.50]	0.001
Muscle mass (kg)	51.10 [46.95, 54.50]	51.50 [46.95, 54.50]	52.10 [48.20, 56.27]	0.033
Basic metabolic rate (kcal/d)	1260.01 ± 128.83	1275.21 ± 125.29	1288.02 ± 137.51	0.033

### 3.2 Comparison of anthropometric, biochemical characteristics, sex hormones, body composition and chronic comorbidities in elderly women stratified by 25-(OH)D levels

In elderly women, as the 25-(OH)D levels decreased across the groups, BMI, WC, and WHR, as well as PTH levels, increased (P<0.05). In contrast, the levels of Ca, HDL-c, Cr, AST, Alb, and Hb decreased (P<0.05). Regarding sex hormones, PRL levels increased gradually, while LH and FSH levels showed a decreasing trend (P<0.05). For body composition indicators, the body fat percentage, muscle mass, and basic metabolic rate increased gradually (P<0.05). Additionally, the proportion of individuals with coronary heart disease increased, as presented in **Table 2**.

**Table 2:** Comparison of key characteristics in elderly women stratified by 25-(OH)D levels.

25-(OH)D	Sufficiency (≥30 ng/ml), n=87	Insufficiency (20~30 ng/ml), n=408	Deficiency (<20 ng/ml), n=571	P-value
Age (ys)	66.00 ± 4.49	65.36 ± 4.82	65.61 ± 4.93	0.503
BMI (Kg/m^2^)	24.05 ± 3.44	24.48 ± 3.08	25.28 ± 3.30	<0.001
WC (cm)	84.69 ± 9.95	85.39 ± 9.29	87.33 ± 9.66	0.002
WHR	0.84 ± 0.05	0.85 ± 0.04	0.86 ± 0.05	0.001
T (nmol/L)	0.21 [0.09,0.46]	0.22 [0.09,0.46]	0.26 [0.09,0.51]	0.219
E_2_ (pmol/L)	18.35 [18.35,20.51]	18.35 [18.35,19.09]	18.35 [18.35,20.82]	0.833
LH (U/L)	27.04 [21.12,33.16]	28.56 [22.14,35.90]	26.30 [20.11, 33.25]	0.002
FSH (U/L)	60.53 [46.70,80.30]	63.56 [50.65,78.29]	57.58 [45.12, 72.74]	<0.001
P (nmol/L)	0.16 [0.16, 0.24]	0.16 [0.16, 0.33]	0.16 [0.16, 0.32]	0.317
PRL (u;g/L)	9.35 [7.21, 11.71]	9.95 [7.14, 15.24]	10.52 [7.14, 16.30]	0.031
Ca (mmol/L)	2.37 ± 0.09	2.35 ± 0.09	2.32 ± 0.10	<0.001
PTH (pg/ml)	37.92 [31.17,50.67]	43.48 [36.09,54.42]	49.04 [38.65,63.41]	<0.001
FPG (mmol/L)	5.51 [5.06,6.01]	5.38 [5.07,5.97]	5.46 [5.06,6.07]	0.752
HbA1c (%)	6.00 [5.70,6.40]	6.00 [5.70,6.30]	6.00 [5.70,6.30]	0.851
TC (mmol/L)	4.89 ± 1.01	5.02 ± 0.97	4.91 ± 1.00	0.212
LDL-C (mmol/L)	3.34 ± 1.02	3.46 ± 1.00	3.37 ± 1.03	0.309
TG (mmol/L)	1.40 [1.06,1.80]	1.37 [1.05,1.77]	1.49 [1.09, 1.99]	0.080
HDL-C (mmol/L)	1.45 [1.21, 1.74]	1.43 [1.20,1.72]	1.32 [1.11, 1.56]	<0.001
UA (mmol/L)	280.50 [250.25,332.50]	284.00 [252.00,330.00]	291.00 [247.50, 328.50]	0.986
Cr (u;mol/L)	58.00 [52.00,66.00]	58.00 [52.00,64.00]	56.00 [50.00,63.00]	0.011
ALT (U/L)	16.20 [12.90,22.15]	16.40 [12.60,21.35]	15.90 [12.30,21.45]	0.614
AST (U/L)	19.45 [16.68,22.78]	18.70 [15.95,21.95]	17.40 [15.00,21.25]	0.001
Alb (g/L)	45.77 ± 3.19	45.48 ± 3.04	44.56 ± 3.29	<0.001
Hb (g/L)	133.48 ± 9.76	132.52 ± 9.52	131.06 ± 9.87	0.017
CRP (mg/dl)	0.10 [0.06,0.17]	0.10 [0.05,0.19]	0.11 [0.06,0.21]	0.168
Diabetes (%)	16 (18.6%)	63 (15.4%)	95 (16.6%)	0.207
Hypertension (%)	22(26.5%)	146 (37.2%)	219 (38.9%)	0.093
CHD (%)	2 (2.3%)	17 (4.2%)	36 (6.3%)	0.042
Body fat percentage (%)	31.50 [29.68, 34.52]	31.80 [29.40, 34.90]	33.60 [30.60, 35.65]	<0.001
Muscle mass (kg)	37.15 [35.08, 40.00]	37.80 [35.53, 40.50]	38.70 [36.00, 41.30]	0.003
Basic metabolic rate (kcal/d)	1036.06 ± 55.71	1043.88 ± 58.31	1050.75 ± 61.62	0.040

### 3.3 The association of 25-(OH)D with sex hormones in elderly men

In elderly men, a 10 ng/ml decrease in 25-(OH)D was associated with an average difference of −0.95 (−1.46, −0.45) nmol/L in testosterone levels; however, this association disappeared after adjusting for age and BMI (**Table 3**). Logistic regression analysis revealed that for every 10 ng/ml decrease in 25-(OH)D, the risk of hypogonadism (defined as total testosterone < 12 nmol/L) increased by 26% (P < 0.05). Nevertheless, this association was no longer statistically significant following adjustment for confounding factors, including age and BMI (**Table 4**).

**Table 3:** The association of 25-(OH)D and sex hormones in elderly men.

Variables	Model 1		Model 2		Model 3		Model 4	
25-(OH)D, per 10 ng/ml lower	β(95%CI)	P-value	β(95%CI)	P-value	β(95%CI)	P-value	β(95%CI)	P-value
T	-0.95(-1.46, -0.45)	0.000	-0.41(-0.88, 0.06)	0.085	-0.57(-1.04, -0.10)	0.018	-0.35(-0.82, 0.12)	0.142
E_2_	-3.39(-6.52, -0.25)	0.034	-0.33(-6.49, -0.16)	0.039	-3.77(-6.96, -0.58)	0.021	-2.49(-5.74, 0.76)	0.133
LH	-0.00(-0.41, 0.41)	0.995	0.23(-0.16, 0.62)	0.238	0.09(-0.30, 0.47)	0.668	0.11(-0.29, 0.51)	0.578
FSH	-0.12(-0.83, 0.59)	0.738	0.231(-0.45, 0.91)	0.504	0.23(-0.46, 0.91)	0.520	0.29(-0.41, 0.10)	0.414
P	-0.01(-0.03, 0.02)	0.416	-0.00(-0.03, 0.02)	0.940	-0.01(-0.04, 0.02)	0.427	-0.00(-0.03, 0.02)	0.894
PRL	0.39(-1.52, 2.29)	0.691	0.46(-1.47, 2.39)	0.642	0.116(-1.83, 2.06)	0.907	0.42(-1.57, 2.42)	0.676

β: Adjusted difference

Model 1: No adjusted factors.

Model 2: Adjusted for age and BMI.

Model 3: Adjusted for age, BMI and Alb.

Model 4: Adjusted for age, BMI, WC, Alb, Ca, AST, Cr, HDL-C, and smoking status.

**Table 4:** Correlation between 25-(OH)D and hypogonadism (total testosterone < 12 nmol/L).

Variables	OR(95%CI)			
	Model 1	Model 2	Model 3	Model 4
25-(OH)D, per 10 ng/ml lower	**1.26(1.04, 1.53)**	1.10 (0.90, 1.35)	1.15 (0.94, 1.42)	1.08 (0.87, 1.33)
25-(OH)D ≥30ng/ml	Reference(1)	Reference(1)	Reference(1)	Reference(1)
25-(OH)D 20–<30 ng/ml	1.29(0.88, 1.94)	1.18(0.79, 1.79)	1.18(0.79, 1.80)	1.16(0.77, 1.78)
25-(OH)D 10–<20 ng/ml	1.44(0.97, 2.18)	1.18(0.78, 1.81)	1.24(0.82, 1.91)	1.11(0.72, 1.73)
25-(OH)D <10 ng/ml	1.57(0.41, 5.11)	1.40(0.35, 4.72)	1.49(0.37, 5.11)	1.32(0.32, 4.87)

Statistically significant results are bolded.

Model 1: No adjusted factors.

Model 2: Adjusted for age and BMI.

Model 3: Adjusted for age, BMI and Alb.

Model 4: Adjusted for age, BMI, WC, Alb, PRL, Ca, AST, Cr, HDL-C, and smoking status.

### 3.4 The association of 25-(OH)D with sex hormones in elderly women

In elderly women, a 10 ng/ml decrease in 25-(OH)D was associated with decreases in LH and FSH levels. Nevertheless, no statistically significant differences were observed after adjusting for confounding factors, including age, BMI, and Alb (**Table 5**).

**Table 5:** The association of 25-(OH)D and sex hormones in elderly women.

Variables	Model 1		Model 2		Model 3		Model 4	
25-(OH)D, per 10 ng/ml lower	β(95%CI)	P-value	β(95%CI)	P-value	β(95%CI)	P-value	β(95%CI)	P-value
T	0.01(-0.03, 0.05)	0.627	0.00(-0.03, 0.04)	0.837	-0.00(-0.04, 0.04)	0.957	0.01(-0.03, 0.05)	0.622
E_2_	0.31(-1.38, 1.99)	0.722	-0.20(-1.88, 1.48)	0.817	-0.27(-1.98, 1.42)	0.749	-0.31(-2.03, 1.41)	0.725
LH	-1.19(-2.22, -0.16)	0.024	-0.77(-1.79, 0.25)	0.136	-0.83(-1.86, 0.20)	0.115	0.05(-0.63, 0.73)	0.881
FSH	-4.05(-6.34, -1.77)	0.001	-2.66(-4.88, -0.43)	0.019	-2.14(-4.38, 0.10)	0.061	-1.63(-3.87, 0.62)	0.157
P	0.01(-0.01, 0.04)	0.409	0.02(-0.01, 0.04)	0.238	0.007(-0.02, 0.03)	0.608	0.01(-0.02, 0.04)	0.418
PRL	0.80(-0.08, 1.68)	0.075	0.96(0.08, 1.85)	0.033	0.27(-0.58, 1.13)	0.529	0.28(-0.58, 1.15)	0.523

25-(OH)D:25-hydroxyvitamin D; T: testosterone; E2: estradiol; P: progesterone; LH: luteinizing hormone; FSH: follicle-stimulating hormone; PRL: prolactin

β: Adjusted difference

Model 1: No adjusted factors.

Model 2: Adjusted for age and BMI.

Model 3: Adjusted for age, BMI and Alb.

Model 4: Adjusted for age, BMI, WC, Alb, Hb, Ca, AST, Cr and HDL-C.

### 3.5 The association of 25-(OH)D with body composition

**Figure 1:**
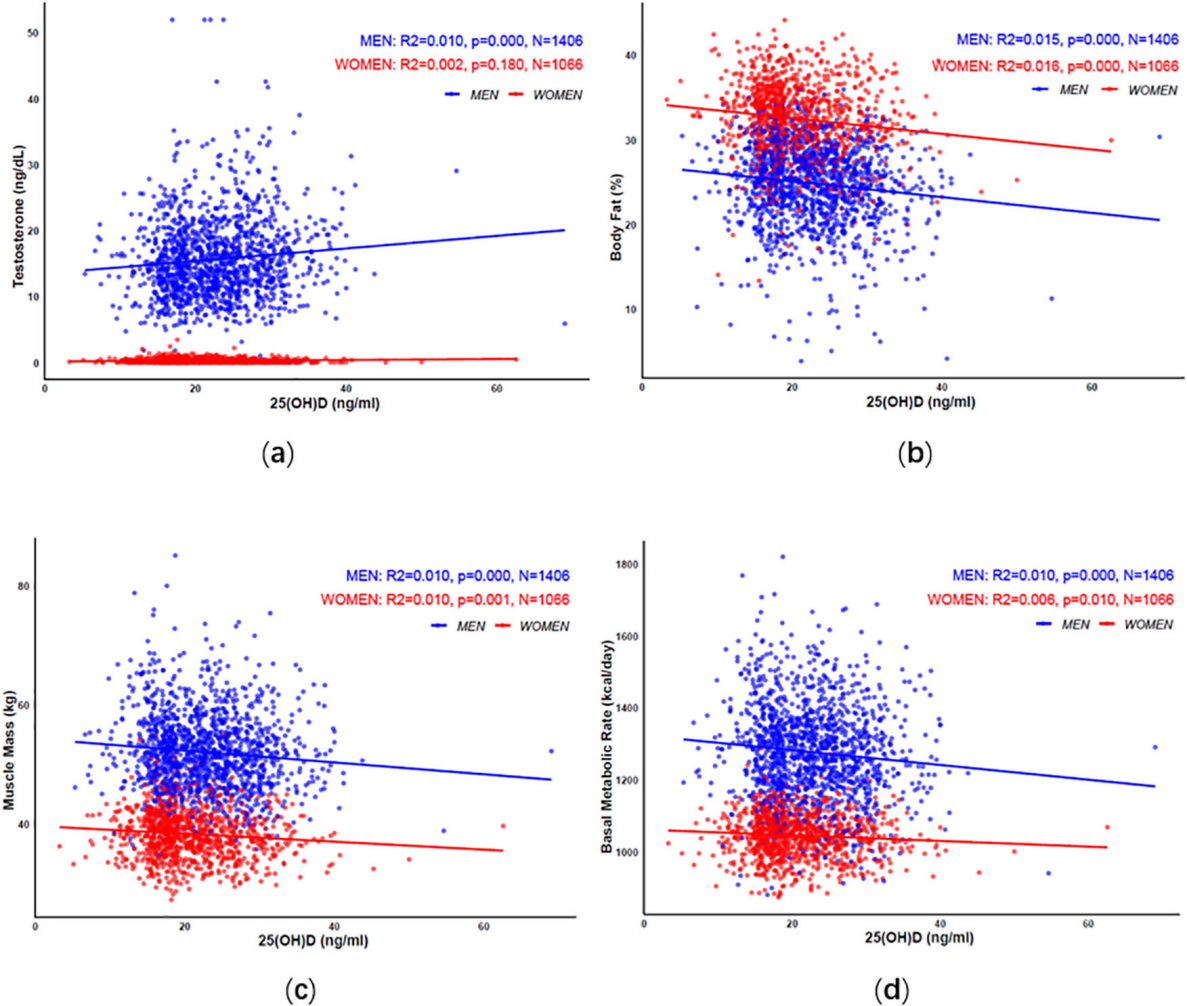
The correlation of vitamin D with testosterone **(a)**, body fat percentage **(b)**, muscle mass **(c)**, and basal metabolic rate **(d)** in elderly men and women.

The correlation coefficient plot showed that in men, vitamin D levels were positively correlated with testosterone, and negatively correlated with body fat percentage, muscle mass, and basal metabolic rate. In women, vitamin D levels were not associated with testosterone but were negatively correlated with body fat percentage, muscle mass, and basal metabolic rate, see **Figure 1a–d**.

For every 10 ng/ml decrease in 25-(OH)D, body fat percentage, muscle mass, and basal metabolic rate increased in both older men and women. After adjusting for confounding factors, only body fat percentage in elderly men remained increased by 0.30% (0.00%, 0.59%), P<0.05, as shown in **Table 6**.

**Table 6:** The association of 25-(OH)D and body composition.

Variables	Model 1		Model 2		Model 3		Model 4	
25-(OH)D, per 10 ng/ml lower	β(95%CI)	P-value	β(95%CI)	P-value	β(95%CI)	P-value	β(95%CI)	P-value
Males								
Body fat percentage	0.93(0.54, 1.32)	0.000	0.19(-0.10, 0.47)	0.198	0.31(0.03, 0.60)	0.033	0.30(0.00, 0.59)	0.047
Muscle mass	1.00(0.47, 1.53)	0.000	-0.02(-0.42, 0.38)	0.908	-0.13(-0.53, 0.27)	0.521	-0.18(-0.58, 0.23)	0.386
Basic metabolic rate	20.49(9.60, 31.39)	0.000	0.50(-7.45, 8.46)	0.901	-1.11(-9.13, 6.90)	0.786	-2.15(-10.16, 5.87)	0.599
Females								
Body fat percentage	0.93(0.49, 1.36)	0.000	0.09(-0.14, 0.33)	0.447	0.13(-0.11, 0.37)	0.288	0.15(-0.09, 0.39)	0.226
Muscle mass	0.67(0.26, 1.07)	0.001	0.07(-0.24, 0.38)	0.655	0.01(-0.31, 0.32)	0.962	-0.02(-0.34, 0.29)	0.885
Basic metabolic rate	7.90(1.90, 13.90)	0.010	1.06(-3.11, 5.23)	0.617	0.28(-3.93, 4.49)	0.895	0.01(-4.20, 4.21)	0.998

β: Adjusted difference

Model 1: No adjusted factors.

Model 2: Adjusted for age and BMI.

Model 3: Adjusted for age, BMI and Alb.

Model 4: Adjusted for age, BMI, WC, Alb, Hb, Ca, AST, Cr and HDL-C.

## 4 Discussion

Vitamin D deficiency represents a significant public health concern globally, particularly among older adult populations. The potential relationship between vitamin D status and sex hormones has garnered scientific interest, particularly given the parallel decline of both vitamin D and sex hormones with advancing age. Testosterone decline in aging men and estrogen reduction in postmenopausal women represent significant endocrine changes that impact multiple health outcomes. Similarly, age-related changes in body composition, including increased adiposity and reduced muscle mass, present substantial health challenges for older adults. Given the significant sex differences in gonadal hormones and body fat composition, this study analyzed the relationships between vitamin D, sex hormones, and body composition separately in elderly male and female populations. We revealed high rates of vitamin D insufficiency, and deficiency among older adults, consistent with global patterns of vitamin D status in elderly populations (3, 25). Among male participants, 87.4% demonstrated insufficient or deficient vitamin D levels, and the prevalence was even higher in female participants, with 91.8% below sufficient levels. Furthermore, we found that in older men, as vitamin D levels decreased, testosterone showed a decreasing trend, but this difference disappeared after adjusting for age and BMI; the body fat percentage increased even after adjusting for confounders.

The relationship between vitamin D status and sex hormones in older individuals is inconsistent currently, as different studies have reported conflicting results. It is hypothesized that vitamin D may modulate sex hormone synthesis by activating vitamin D receptors (VDRs) in gonadal tissues, which in turn regulate the expression of key enzymes (e.g., cholesterol side-chain cleavage enzyme) involved in the biosynthesis of testosterone and estrogen (7, 8, 26). A study of older Dutch men (65–89 years) found that serum 25-(OH)D was positively associated with total testosterone levels, and this association remained significant after adjustments for confounders (27). In a study of middle-aged and older Chinese men, a lower vitamin D level was associated with a higher prevalence of hypogonadism, but it is worth noting that this association was considerably attenuated by BMI and HOMA-IR (11). Moreover, research in Malaysian men demonstrated that 25-(OH)D was significant positively associated with total testosterone, but this association was BMI-dependent (28). Similarly, in our study, we found that a 10 ng/ml decrease in 25-(OH)D was associated with an average decrease of 0.95 nmol/L in testosterone levels, but this association was attenuated after adjusting for age and BMI. Since there was no age difference between the different 25-(OH)D stratification groups, we consider BMI to be the main confounding factor. Therefore, we speculate that in the elderly men, BMI is a key factor influencing vitamin D deficiency-related hypogonadism.

The relationship between vitamin D and estradiol in older women appears complex and inconsistent across studies. The present study found no association between 25-(OH)D status and estradiol, which is in line with a study of postmenopausal women found no significant correlation between estradiol levels and vitamin D levels (29). Conversely, other researches have reported a positive correlation between estradiol and 25-(OH)D (30, 31). This discrepancy may be explained by population differences, methodological variations, or the complex interplay between menopause, aging, and vitamin D metabolism.

Furthermore, we investigated the association of 25-(OH)D with body composition in elderly individuals. Body fat percentage directly quantifies the proportion of fat tissue in the body. Elderly populations are prone to age-related fat accumulation. Muscle mass represents the total amount of skeletal muscle, a key marker of “sarcopenia”- a common geriatric condition characterized by age-related muscle loss (32). Basic metabolic rate reflects the baseline energy expenditure of the human body at rest, and is inherently determined by body fat percentage and muscle mass. Therefore, in the present study, we use these three indicators to reflect key age-related body composition changes in older adults. We found that body fat percentage, muscle mass and basic metabolic rate showed an increasing trend as vitamin D levels decreased across deficiency categories. After adjusting for confounding factors, only body fat percentage increased in older males. Similarly, Lech et al. demonstrated vitamin D insufficiency was significantly more prevalent (over two times higher odds) among obese community-dwelling older individuals in Poland (33). Several mechanisms may explain the observed relationships between vitamin D status and body fat: First, vitamin D receptors are expressed in adipocytes, and vitamin D may influence adipocyte differentiation and metabolism. Lower vitamin D status may promote adipogenesis and fat accumulation (18, 22, 34); second, adipose tissue is thought to act as a “storage depot” for vitamin D, sequestering it and reducing circulating active vitamin D concentrations (35); third, vitamin D deficiency may reduce mobility and physical activity levels, potentially leading to increased adiposity and muscle loss (36, 37).

Additionally, serum 25(OH)D-deficiency individuals were proven to be more prone to have metabolic syndrome and specific components (38, 39). Our study demonstrated that, in both older males and females, as vitamin D levels decreased across deficiency categories, BMI, WC and WHR increased, while HDL-C declined. These findings are consistent with recent epidemiological evidence. A cross-sectional research involving 1,177 Chinese participants aged 18–90 years has reported that individuals with low 25(OH)D have significantly higher WC, BMI, and WHR, as well as lower HDL−C, compared with those with sufficient vitamin D (40). Moreover, research focusing on older adults revealed that vitamin D deficiency is strongly linked to increased visceral adiposity and central obesity, with WHR exhibiting the strongest association in both sexes (41, 42). Therefore, we can conclude that vitamin D deficiency is closely associated with adiposity and related metabolic disorders.

There are also some limitations in this study. The cross-sectional study design prevents the establishment of the temporal sequence and causal relationship between 25-(OH)D and sex hormones; only total testosterone levels were measured; free testosterone, sex hormone-binding globulin (SHBG), and other related indices were not assessed; 25-(OH)D and sex hormones were measured only once, which is subject to intra-individual variation, measurement error and seasonal effects, and thus may not reflect long-term levels.

## 5 Conclusions

This study demonstrates that vitamin D deficiency is highly prevalent among older adults. In older men, vitamin D status positively correlates with total testosterone; however, this correlation disappeared after adjusting for BMI. Moreover, as vitamin D levels decreased across deficiency categories, WC, WHR, and body fat percentage increased. These data do not support our previous hypothesis, but suggest that increased BMI may play a potential mediating role in the relationship between vitamin D and testosterone. Targeting BMI/adiposity (e.g., via lifestyle interventions to reduce excess body fat) may help mitigate the decline in testosterone levels associated with vitamin D deficiency, thereby improving overall quality of life in aging males.

## Data Availability

The raw data supporting the conclusions of this article will be made available by the authors, without undue reservation.

## Supplementary material

Supplementary Figure 1 Flowchart illustrating the inclusion/exclusion of individuals in this study.
