# Supplementary figures and images for: Association of 25-hydroxyvitamin D with sex hormones and body composition in Chinese older adults
Source: Front Endocrinol (Lausanne). 2025 Nov 17;16:1714445. doi: 10.3389/fendo.2025.1714445 (PMC12665557; doi:10.3389/fendo.2025.1714445)

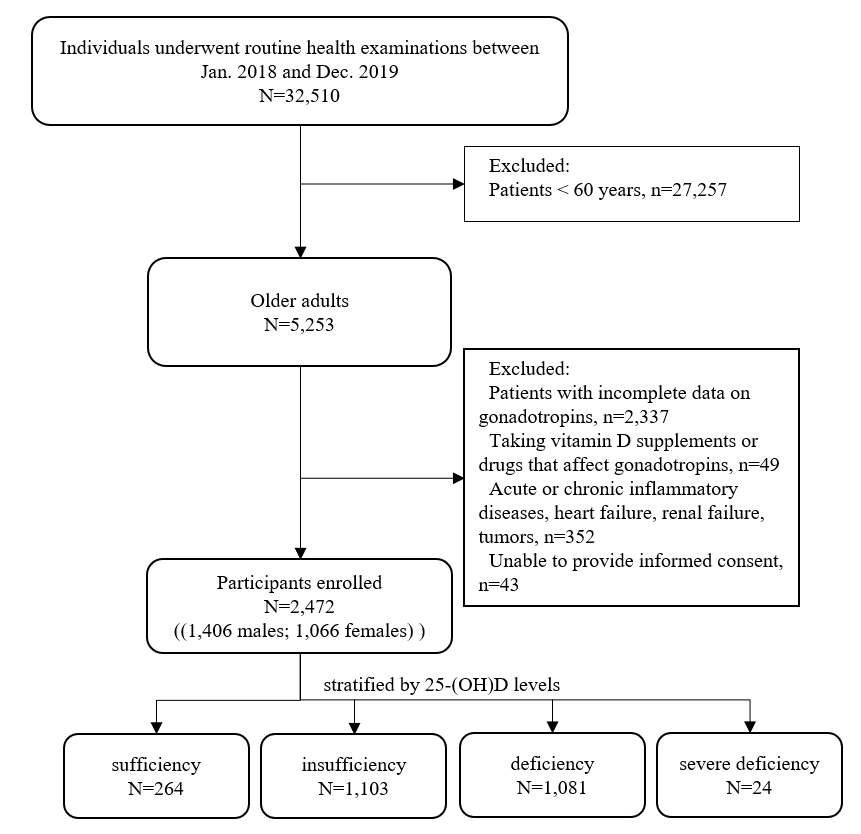

Supplement: Supplementary Figure 1 — Flowchart illustrating the inclusion/exclusion of individuals in this study. [file Image1.tiff]
